# Comparative analysis of romosozumab and denosumab treatment in hemodialysis patients with osteoporosis: a 12-mo observational study

**DOI:** 10.1093/jbmrpl/ziaf096

**Published:** 2025-06-04

**Authors:** Masatoshi Teraguchi, Yorihide Kitayama, Yuki Yamada, Zyun Nakamoto, Yoshiyuki Yamaoka, Hiroshi Yamada

**Affiliations:** Department of Orthopaedic Surgery, Wakayama Medical University, Wakayama 641-8509, Japan; Chuki Clinic, Wakayama 649-1342, Japan; Chuki Clinic, Wakayama 649-1342, Japan; Chuki Clinic, Wakayama 649-1342, Japan; Chuki Clinic, Wakayama 649-1342, Japan; Department of Orthopaedic Surgery, Wakayama Medical University, Wakayama 641-8509, Japan

**Keywords:** hemodialysis, osteoporosis, romosozumab, denosumab, bone turnover markers

## Abstract

This study aimed to evaluate and compare the efficacy and safety of romosozumab vs denosumab in hemodialysis (HD) patients with osteoporosis. A 12-mo observational study was conducted involving HD patients with osteoporosis at a single dialysis center. The study compared outcomes between 2 treatment groups: romosozumab (210 mg monthly, *n* = 21) and denosumab (60 mg every 6 mo, *n* = 24). Treatment allocation was based on cardiovascular history. Changes in bone mineral density (BMD), bone turnover markers (P1NP, TRACP-5b, and intact PTH), complications, and fractures were assessed. Serum calcium levels were monitored weekly. After 12 mo, patients receiving romosozumab showed significantly greater improvements in lumbar spine (LS) BMD compared to denosumab (14.6% vs 6.3%, *p* < .05). Both treatments demonstrated comparable increases in femoral neck (FN) BMD (4.3% vs 6.0%, *p* = .41). Bone turnover markers showed distinct patterns between groups, with romosozumab producing more pronounced early effects. P1NP levels increased significantly in the romosozumab group at 6 mo (+41.2%) before declining toward baseline, while showing sustained suppression in the denosumab group. The incidence of osteoporotic fractures was one case per group (vertebral fracture in romosozumab group, hip fracture in denosumab group). One case of injection site reactions was observed in the romosozumab group. Mean serum calcium levels remained stable in both groups throughout the study period. The treatment of romosozumab appears to be more effective than denosumab for improving BMD in HD patients with osteoporosis, particularly at the LS. Both treatment approaches demonstrated acceptable safety profiles, though careful monitoring of calcium levels and cardiovascular status is recommended.

## Graphical Abstract

**Graphical Abstract:**
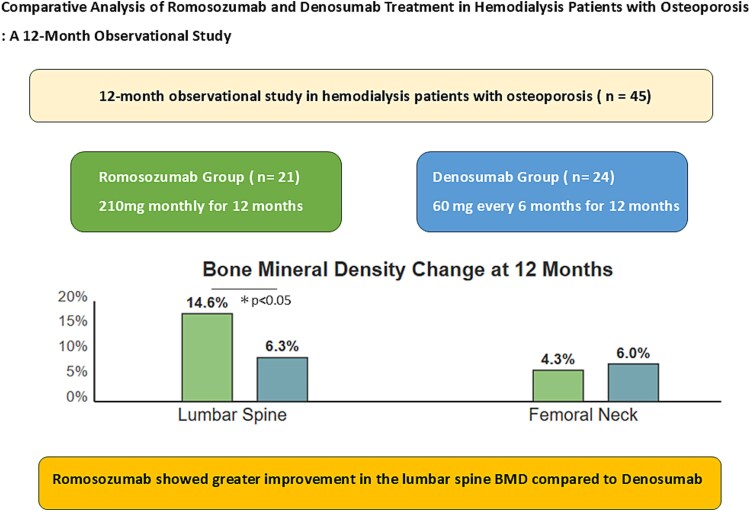

## Introduction

Chronic kidney disease-mineral bone disorder (CKD-MBD) represents a systemic condition that develops as a consequence of CKD, characterized by abnormalities in bone and mineral metabolism, with or without vascular calcification.^1^ In hemodialysis (HD) patients, the management of bone disorders is particularly challenging due to the complex interplay between traditional osteoporosis and CKD-MBD, leading to increased fracture risk and subsequent mortality.^2^^,^^3^

The term CKD-MBD was introduced to describe a broader clinical syndrome that encompasses biochemical abnormalities, bone disease, and extraosseous calcification.^1^^,^^4^ In HD patients, bone fragility represents a significant clinical challenge, with fracture rates reported to be 4-fold higher than in the general population.^5^ Moreover, the post-fracture mortality rate in HD patients is substantially higher compared to non-HD patients, emphasizing the critical need for effective therapeutic interventions.^6^

Historically, the management of bone disease in HD patients has focused primarily on controlling secondary hyperparathyroidism through the use of vitamin D receptor activators (VDRAs) and calcimimetics.^7^ However, this approach alone has proven insufficient for preventing fractures, particularly in patients with concurrent osteoporosis. The use of traditional osteoporosis medications in HD patients has been limited by concerns about safety and efficacy in the setting of altered bone metabolism.^8^

Bisphosphonates, while effective in the general population, have been used cautiously in HD patients due to concerns about oversuppression of bone turnover and long-term accumulation.^8^^,^^9^ These concerns are particularly relevant given the high prevalence of adynamic bone disease in the HD patients. Additionally, the reduced renal clearance of bisphosphonates in end-stage renal disease has led to limited data on their safety and efficacy in this population.^10^

The introduction of denosumab, a fully human monoclonal antibody against Receptor Activator of Nuclear factor Kappa-B Ligand (RANKL), represented a significant advance in the treatment options for HD patients with osteoporosis. Unlike bisphosphonates, denosumab is not cleared by the kidneys, and its pharmacokinetics are not significantly altered in renal impairment.^11^ Several studies have demonstrated the efficacy of denosumab in improving BMD in HD patients, although concerns about severe hypocalcemia have necessitated careful monitoring and supplementation protocols.^11^^,^^12^

More recently, the development of romosozumab, a monoclonal antibody targeting sclerostin, has opened new therapeutic possibilities. Sclerostin, a glycoprotein produced by osteocytes, is a key inhibitor of bone formation through its effects on the Wnt/β-catenin signaling pathway.^13^ Interestingly, sclerostin levels are typically elevated in HD patients, potentially contributing to their bone fragility.^14^ This observation has led to particular interest in sclerostin inhibition as a therapeutic strategy in this population. The mechanism of action of romosozumab differs fundamentally from previous osteoporosis treatments through its dual effect of increasing bone formation while decreasing bone resorption.^15^ Clinical studies in postmenopausal women have demonstrated superior BMD gains with romosozumab compared to both placebo and active comparators.^16^ However, the optimal use of romosozumab in HD patients, particularly in sequence with other osteoporosis medications, remains to be fully elucidated.

The complex nature of bone disease in HD patients necessitates careful consideration of treatment strategies. The altered mineral metabolism, presence of vascular calcification, and increased risk of hypocalcemia all present unique challenges in this population.^17^ Furthermore, the interpretation of traditional markers of bone turnover is complicated by their altered clearance in renal failure, making monitoring of treatment response more challenging.^18^

Given these considerations, there is a clear need for evidence regarding the optimal approach to treating osteoporosis in HD patients. The potential for sequential therapy, utilizing agents with complementary mechanisms of action, represents an attractive therapeutic strategy that requires further investigation. Understanding the relative efficacy and safety of different treatment approaches, particularly the role of newer agents, such as romosozumab, is crucial for improving outcomes in this high-risk population.

The present study was designed to address these knowledge gaps by comparing the efficacy of romosozumab vs denosumab in HD patients with osteoporosis. Through comprehensive evaluation of BMD changes, bone turnover markers, and safety parameters, we aimed to provide insights that would help guide clinical decision-making in this challenging patient population.

## Methods

**Figure 1:**
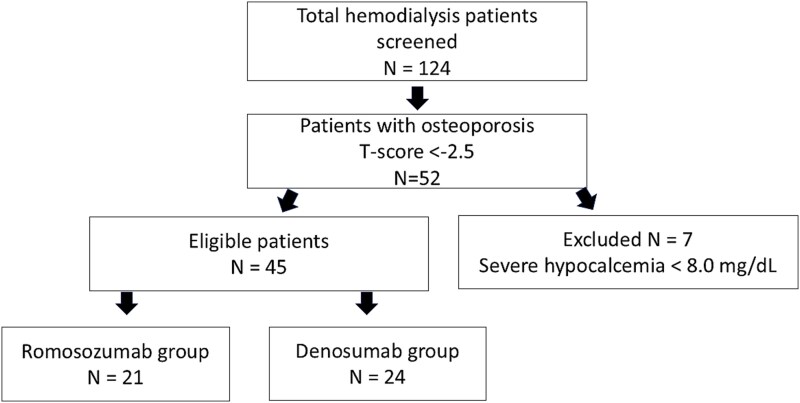
Flowchart of patient inclusion. From a total of 124 patients receiving maintenance hemodialysis at our center, we identified 52 patients with osteoporosis. Osteoporosis was defined according to WHO criteria as a T-score of less than −2.5 at any major skeletal site (LS and FN). Seven patients were excluded, due to severe hypocalcemia (serum calcium <8.0 mg/dL despite calcium supplementation). The remaining 45 patients were enrolled in the study.

This prospective observational study was conducted at one dialysis center between November 2019 and March 2022. From a total of 124 patients receiving maintenance HD at our center, we identified 52 patients with osteoporosis. Osteoporosis was defined according to WHO criteria as a T-score of less than −2.5 at any major skeletal site (LS and FN). Seven patients were excluded due to severe hypocalcemia (serum calcium < 8.0 mg/dL despite calcium supplementation). The remaining 45 patients were enrolled in the study. Additional exclusion criteria included recent cardiovascular events within 1 yr, pathological fracture, active malignancy, or inability to provide informed consent (Figure 1).

Treatment allocation was determined based on cardiovascular history. Patients with a history of cardiovascular events were preferentially assigned to the denosumab group, while those without cardiovascular events were generally allocated to receive romosozumab. Cardiovascular events were defined as previous myocardial infarction or stroke requiring intervention.

Patients in the romosozumab group received monthly subcutaneous injections of romosozumab (210 mg) for 12 mo. The denosumab group received subcutaneous injections of denosumab (60 mg) every 6 mo for 12 mo. All patients continued their standard dialysis regimens (either conventional HD or hemodiafiltration [HDF]) and received calcium and vitamin D supplementation as clinically indicated.

All patients received calcium carbonate supplementation (1000-1500 mg elemental calcium daily, adjusted based on serum calcium levels) and alfacalcidol (0.25-0.5 μg daily). Calcium levels were monitored weekly for the first month after drug administration, then biweekly for the second month, and monthly thereafter. If serum calcium levels decreased below 8.5 mg/dL, calcium carbonate dosage was increased by 500 mg, and if levels fell below 8.0 mg/dL, alfacalcidol dosage was increased by 0.25 μg with twice-weekly monitoring until normalization.

We assessed BMD using DXA at baseline, 6-mo, and 12-mo throughout the study period. Measurements were taken at the LS (L1-4) and FN. Bone turnover was monitored through serum levels of total P1NP (bone formation marker), TRACP-5b (bone resorption marker), and intact PTH at baseline, 6 and 12 mo. Serum calcium levels were monitored every 2 wk during the first month after each drug administration, then biweekly for the second month, and monthly thereafter. The 12-mo incidence of osteoporotic fractures and treatment-related complications were recorded.

### Statistical analysis

Changes in BMD and bone turnover markers were analyzed using mixed-effects models with treatment group, time, and their interaction as fixed effects, and patient as a random effect. Due to our small sample size and after confirming non-normal distribution of several variables using the Shapiro–Wilk test, we employed the Mann–Whitney U test for comparing continuous variables between groups. For categorical variables, we used Fisher’s exact test. We calculated standardized mean differences (SMDs) for all baseline characteristics to better assess between-group balance. *p*-values <.05 were considered statistically significant. All analyses were performed using JMP version 16.1 (SAS Institute).

To control for potential confounding factors, we compared baseline characteristics between groups using SMD in addition to statistical tests. SMD of ≥0.2, ≥0.5, and ≥0.8 were considered to represent small, moderate, and large differences, respectively. Although treatment allocation was based on cardiovascular history, the baseline characteristics analysis suggested comparable groups despite this non-randomized assignment. In this study, there were no missing data for the primary outcome measures as all 45 enrolled patients completed the 12-mo follow-up with all scheduled DXA scans and laboratory assessments. Had missing data occurred, we planned to use the last observation carried forward method for primary analyses and conduct sensitivity analyses using multiple imputation techniques.

### Sample size determination

The sample size for this study was determined based on a priori power analysis. The sample size for this study was determined based on a priori power analysis using data from Inose et al.^19^ Based on their reported BMD changes with romosozumab in HD patients and comparative data for denosumab from Iseri et al.,^20^ we calculated that at least 20 patients in each group would be needed to detect this difference with 80% power and a significance level of 0.05. Accounting for potential dropouts, we set the target enrollment at a minimum of 20 patients per group, ultimately enrolling 45 patients (21 in the romosozumab group and 24 in the denosumab group). This sample size was considered sufficient to detect statistically significant differences in BMD changes between groups with 80% power and a significance level of 0.05. It should be noted that the study was conducted at a single dialysis center, and all patients meeting the eligibility criteria were enrolled, which also influenced the sample size determination.

## Results

A total of 45 patients completed the study (21 in the romosozumab group and 24 in the denosumab group). Baseline characteristics were comparable between groups (Table 1). Mean ages were 70.9 ± 10.0 yr in the romosozumab group and 75.8 ± 10.3 yr in the denosumab group. The proportion of female patients was identical (66.7%), as was the mean duration of dialysis (113.9 ± 81.6 vs 152.1 ± 129.8 mo). HD duration, Kt/V, dialysis modalities, comorbidity, pre-existing fractures, causative diseases for HD, previous osteoporosis medications, BMD and baseline laboratory values were similar between groups. One patient in the romosozumab group had previously received bisphosphonate. Overall, most baseline characteristics showed SMDs values below the commonly accepted threshold of 0.5, indicating relatively balanced groups. Despite these observed imbalances, none of the baseline characteristics showed statistically significant differences between treatment groups (all *p*-values > .05), suggesting that randomization achieved relatively comparable groups for the primary analysis (Table 1).

**Table 1:** Baseline clinical characteristics of hemodialysis patients treated by romosozumab or denosumab.

	**Romosozumab**	**Demosumab**	***p*-value**	**SMD**
***N***	21	24		
**Age (yr)**	70.9 (10.0)	75.8 (10.3)	0.12	−0.48
**Gender (m/f)**	7. 14	8. 16	1	−0.02
**BMI (kg/m** ^**2**^**)**	20.4 (3.4)	20.4 (3.1)	0.99	0
**Hemodialysis parameters**				
**HD duration**	113.9 (81.6)	152.1 (129.8)	0.25	−0.35
**Kt/V**	1.60 (0.3)	1.67 (0.3)	0.48	−0.23
**HD/HDF**	11 10	11 13	0.77	−0.09
**Comorbidity**				
**Coronary ischemic disease**	2(9.5%)	5(20.8)	0.3	−0.32
**Pre-existing fractures, *n* (%)**				
**Vertebral, *n* (%)**	1 (4.8%)	2 (8.3%)	0.2	0.18
**Hip, *n* (%)**	0 (0%)	0 (0%)		
**Other, *n* (%)**	1 (4.8%)	1 (4.2%)		
**Causative diseases for hemodialysis**				
**Diabetic nephropathy, *n* (%)**	8 (38.1%)	8 (33.3%)	0.37	0.1
**Chronic glomerulonephritis, *n* (%)**	2 (9.5%)	7 (29.2%)		−0.51
**Nephrosclerosis, *n* (%)**	4 (19.0%)	3 (12.5%)		0.18
**IgA nephropathy, *n* (%)**	1 (4.8%)	0 (0%)		0.32
**Others, *n* (%)**	6 (28.6%)	6 (25.0%)		0.08
**Previous osteoporosis medications**				
**Bisphosphonates, *n* (%)**	1 (4.8%)	0 (0%)	0.28	0.32
**None, *n* (%)**	20 (95.2%)	24 (100%)		−0.32
**Laboratory parameters**				
**Serum Ca (mg/dL)**	8.6 (0.7)	8.8 (0.5)	0.5	−0.33
**Serum P (mg/dL)**	4.7 (1.2)	4.7 (1.0)	0.99	0
**Serum Alb (g/dL)**	3.6 (0.3)	3.6 (0.3)	0.9	0
**Serum e-GFR**	4.5 (1.3)	4.8 (1.5)	0.46	−0.21
**Serum total P1NP (ng/mL)**	314.1 (205.8)	281.7 (244.9)	0.64	0.14
**Serum TRACP-5b (mU/dL)**	548.8 (357.3)	659.2 (349.9)	0.31	−0.31
**Serum i PTH (pg/mL)**	135.8 (84.2)	145.9 (106.3)	0.73	−0.11
**BMD**				
**L1-4 BMD (g/cm** ^**2**^**)**	1.02 (0.37)	0.93 (0.24)	0.32	0.29
**L1-4 T-score**	−0.36 (2.96)	−1.10 (1.89)	0.33	−0.31
**L1-4 Z-score**	0.71 (2.80)	0.26 (1.96)	0.53	−0.19
**Femur neck BMD (g/cm** ^**2**^**)**	0.65 (0.13)	0.61 (0.18)	0.47	0.25
**Femur neck T-score**	−2.68 (1.13)	−3.00 (1.64)	0.46	−0.23
**Femur neck Z-score**	−1.26 (1.39)	−1.29 (1.80)	0.95	−0.02

Abbreviations: Alb, albumin; Ca, calcium; HD, hemodialysis; HDF, hemodiafiltration; iPTH, intact parathyroid hormone; PINP, procollagen type-I N propeptide; SMD, standardized mean differences; TRAP-5b, tartrate-resistant acid phosphatase 5b.

Mixed-effects model analysis confirmed significant increases in LS BMD over time in both groups (*p* < .001). At 6 mo, the romosozumab group demonstrated an increase of 9.3 ± 8.3% and the denosumab group showed an increase of 5.7 ± 8.6% from baseline (*p* = .22). At 12 mo, the romosozumab group showed a mean increase of 14.6 ± 9.2% from baseline while the denosumab group showed a 6.3 ± 7.8% increase (*p* < .05). The interaction between treatment and time was significant (*p* < .05), indicating a greater effect with romosozumab compared to denosumab at the LS.

**Figure 2:**
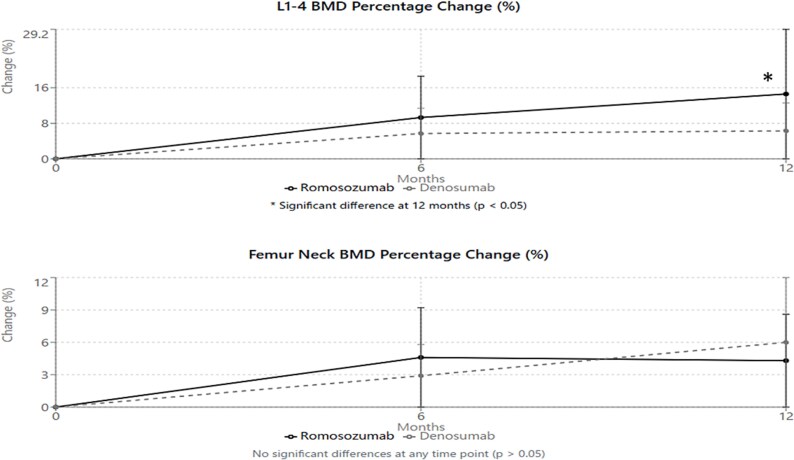
Changes in BMD. At 12 mo, the romosozumab group showed a mean increase of 14.6 ± 9.2% from baseline while the denosumab group showed a 6.3 ± 7.8% increase (*p* < .05).

Improvements in FN BMD were similar between the romosozumab and denosumab groups at both 6 mo (4.6 ± 7.7% vs 2.9 ± 5.2%, *p* = .43) and 12 mo (4.3 ± 7.7% vs 6.0 ± 6.5%, *p* = .41), with no statistically significant differences between groups (Figure 2).

**Figure 3:**
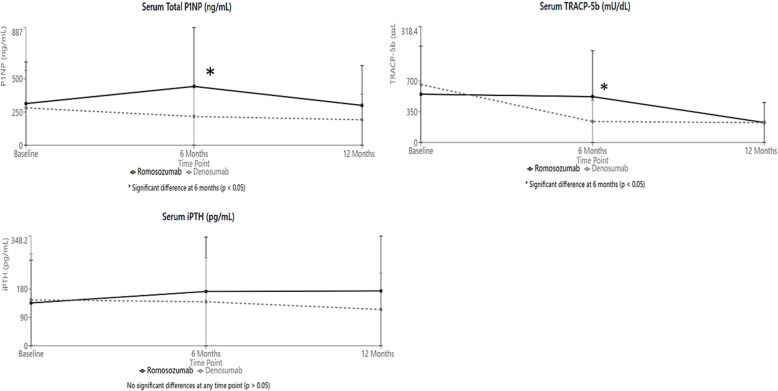
Changes in bone metabolism markers. At 6 mo, the bone formation marker P1NP demonstrated a significant increase in the romosozumab group compared to the denosumab group (443.5 ± 356.8 ng/mL vs 216.9 ± 164.3 ng/mL; *p* < .05). The denosumab group exhibited a more pronounced decrease (238.3 ± 239.3 mU/dL) compared to the romosozumab group (522.1 ± 402.3 mU/dL; *p* < .05).

As shown in Figure 3, bone turnover markers exhibited distinct patterns between the treatment groups over the 12-mo observation period. At baseline, serum total P1NP levels were comparable between groups (romosozumab: 314.1 ± 205.8 ng/mL vs denosumab: 281.7 ± 244.9 ng/mL; *p* = .64). At 6 mo, the bone formation marker P1NP demonstrated a significant increase in the romosozumab group compared to the denosumab group (443.5 ± 356.8 ng/mL vs 216.9 ± 164.3 ng/mL; *p* < .05), followed by a decrease at 12 mo (300.4 ± 218.3 ng/mL vs 192.2 ± 168.7 ng/mL; *p* = .07).

The bone resorption marker TRACP-5b showed similar baseline values (romosozumab: 548.8 ± 357.3 mU/dL vs denosumab: 659.2 ± 349.9 mU/dL; *p* = .31), but demonstrated a significant difference between groups at 6 mo, with the denosumab group exhibiting a more pronounced decrease (238.3 ± 239.3 mU/dL) compared to the romosozumab group (522.1 ± 402.3 mU/dL; *p* < .05). By 12 mo, TRACP-5b levels were nearly identical between groups (romosozumab: 227.3 ± 186.7 mU/dL vs denosumab: 225.0 ± 261.9 mU/dL; *p* = .97).

Intact PTH levels showed divergent trends, although differences did not reach statistical significance. In the romosozumab group, intact PTH increased from baseline (135.8 ± 84.2 pg/mL) to 6 mo (172.4 ± 91.0 pg/mL) and remained elevated at 12 mo (174.1 ± 107.5 pg/mL). In contrast, the denosumab group showed a gradual decrease from baseline (145.9 ± 106.3 pg/mL) to 12 mo (115.3 ± 105.8 pg/mL; *p* = .12 between groups at 12 mo). During the study period, mean serum calcium levels remained stable in both groups (romosozumab: 9.1 ± 0.4 to 9.0 ± 0.5 mg/dL; denosumab: 9.2 ± 0.5 to 9.1 ± 0.4 mg/dL). Weekly serum calcium levels during the first month after medication administration are presented in Figure S1. In the romosozumab group, there was a modest decrease in mean serum calcium from baseline (8.6 ± 0.7 mg/dL) to week 1-2 (8.4 ± 0.5 mg/dL), followed by week 3-4 (8.5 ± 0.4 mg/dL) and 8.4 ± 0.5 mg/dL at week 4. In the denosumab group, a similar pattern was observed, there was a modest decrease in mean serum calcium from baseline (8.8 ± 0.5 mg/dL) with a nadir at week 1-2 (8.3 ± 1.0 mg/dL) followed by week 3-4 (8.7 ± 0.9 mg/dL) and 8.8 ± 0.9 mg/dL at week 4. No patients in either group experienced severe hypocalcemia (defined as serum calcium <8.0 mg/dL) during this period, supporting the effectiveness of our calcium monitoring and supplementation protocol or new cardiovascular events during the treatment period.

One vertebral fracture occurred in the romosozumab group, and one hip fracture occurred in the denosumab group during the 12-mo follow-up period. One case of injection site reaction was observed in the romosozumab group, which resolved without intervention.

## Discussion

Our findings demonstrate that both romosozumab and denosumab can effectively improve BMD in HD patients with osteoporosis. The marked increase in LS BMD observed in the romosozumab group (+14.6 ± 9.2%) is particularly noteworthy, as it exceeds the improvements typically observed with antiresorptive agents alone in HD patients. These results are consistent with the findings from the FRAME study in postmenopausal women, where romosozumab demonstrated significant BMD gains.^16^

The differential response observed between skeletal sites deserves special attention. The more pronounced improvement in LS BMD compared to FN BMD likely reflects the greater proportion of trabecular bone at the spine, which is more responsive to anabolic stimulation.^13^ This site-specific response pattern mirrors findings from previous studies of romosozumab in non-HD patients,^16^ suggesting that the drug’s mechanism of action is preserved in end-stage renal disease. The relatively modest improvements in FN BMD may be attributed to the different composition and metabolic activity of cortical bone, which predominates at this site.

The dynamics of bone turnover markers in our study provide important mechanistic insights. The marked increase in P1NP levels (+41.2% at 6 mo) in the romosozumab group demonstrates the anabolic effect of sclerostin inhibition, suggesting that this mechanism remains effective despite the complex alterations in bone metabolism associated with CKD-MBD.^14^ This finding is particularly significant as elevated sclerostin levels in HD patients often correlate with suppressed bone formation.^21^^,^^22^ Interestingly, our study found that intact PTH levels decreased slightly in the denosumab group, which contrasts with previous findings in the general population where denosumab administration typically leads to increased PTH levels.^23^ This discrepancy may be attributed to the complex mineral metabolism alterations in HD patients, where PTH regulation is already significantly disturbed due to CKD-MBD. Additionally, the concurrent use of vitamin D supplements and calcimimetics in our study population may have mitigated the expected PTH increase. Furthermore, the unique baseline characteristics of our HD population, with generally elevated PTH levels pre-treatment, may have influenced this response pattern.

The stability of serum calcium levels throughout the study period in both groups (romosozumab: 9.1 ± 0.4 to 9.0 ± 0.5 mg/dL; denosumab: 9.2 ± 0.5 to 9.1 ± 0.4 mg/dL) demonstrates the safety of both treatments when accompanied by appropriate monitoring and supplementation protocols. This is particularly noteworthy given previous reports of hypocalcemia in HD patients receiving these medications.

The occurrence of one fracture in each group (vertebral in romosozumab, hip in denosumab) during the 12-mo follow-up period suggests comparable anti-fracture efficacy, though the study was not powered to detect differences in fracture rates. These observations are consistent with previous research demonstrating significant fracture risk reduction with both agents.^16^

Both treatments were generally well-tolerated, with only one case of injection site reaction reported in the romosozumab group. The absence of severe hypocalcemia events reflects the effectiveness of our calcium monitoring and supplementation protocol. The stability of other laboratory parameters, including phosphate and PTH levels, suggests that both treatments can be safely integrated into existing CKD-MBD management protocols.

Our study has several important limitations. First, the analysis using SMD revealed baseline imbalances between treatment groups that merit consideration when interpreting results. Furthermore, small samples may still affect results despite statistical adjustment. Second, the non-randomized treatment allocation based on cardiovascular history represents a significant limitation. While this approach reflects real-world clinical practice, it may introduce selection bias. Groups were not perfectly balanced given the observational nature of the study, with treatment allocation determined by clinical factors rather than randomization. This imbalance is an inherent limitation of our observational design. Even though most standardized differences were below the commonly accepted threshold of 0.1, the absence of randomization means that we cannot fully exclude the influence of selection bias and confounding factors on our outcomes. Our findings should be interpreted as suggesting potential benefits of both treatments in this population rather than establishing superiority of one over the other. Third, the relatively small sample size and single-center nature of the study may limit the generalizability of our findings. Fourth, the 12-mo follow-up period, while sufficient to demonstrate effects on BMD and bone turnover markers, may not be long enough to fully assess fracture risk reduction. Finally, the optimal duration of therapy and long-term safety profiles of these agents in HD patients remain to be determined.

## Conclusions

In this observational study, both romosozumab and denosumab demonstrated effectiveness in improving BMD in HD patients with osteoporosis, with romosozumab showing particularly robust effects at the LS. Both treatments demonstrated acceptable safety profiles when accompanied by appropriate monitoring protocols. Future randomized controlled trials with larger patient populations and longer follow-up periods are needed to establish the comparative efficacy and optimal therapeutic approach for osteoporosis management in HD patients.

## Supplementary Material

Supplementary_figure_ziaf096

## Data Availability

The data that support the findings of this study are available from the corresponding author, upon reasonable request.
