# Supplementary figures and images for: Comparative analysis of romosozumab and denosumab treatment in hemodialysis patients with osteoporosis: a 12-mo observational study
Source: JBMR Plus. 2025 Jun 4;9(8):ziaf096. doi: 10.1093/jbmrpl/ziaf096 (PMC12256106; doi:10.1093/jbmrpl/ziaf096)

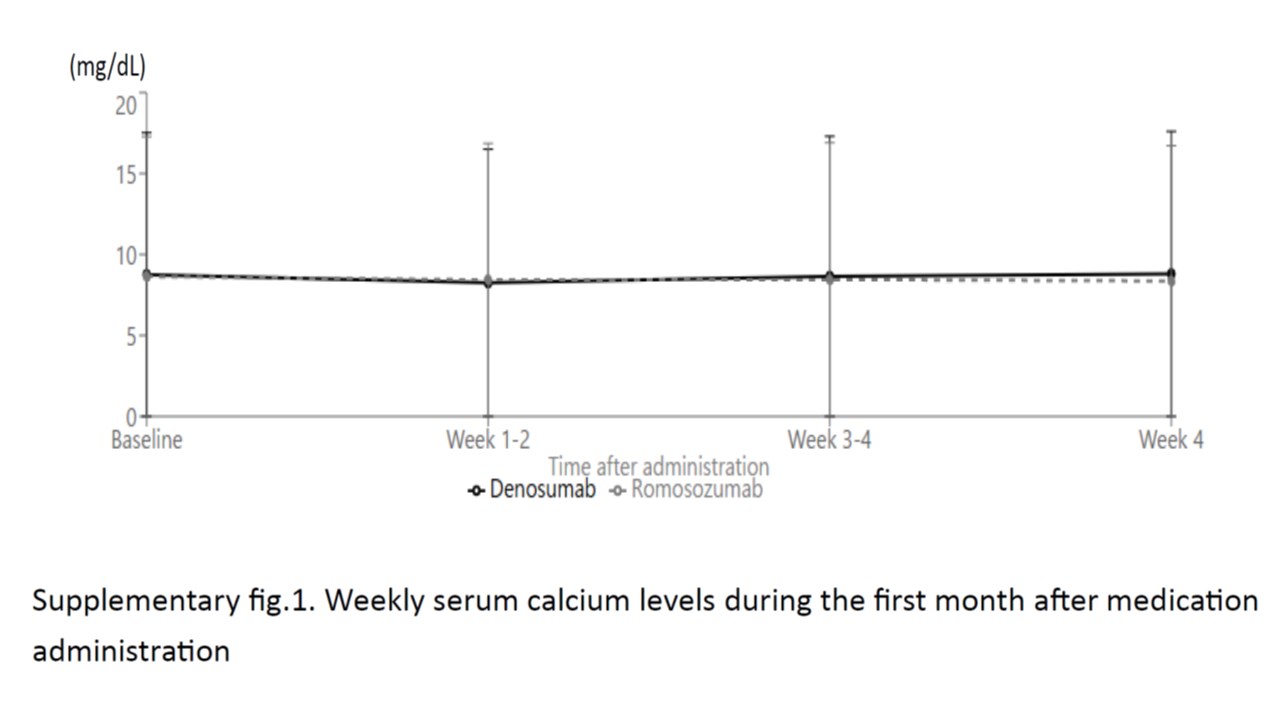

Supplement: Supplementary_figure_ziaf096 [file supplementary_figure_ziaf096.jpeg]
